# The ATP-binding cassette transporters ABCB1 and ABCC1 are not regulated by hypoxia in immortalised human brain microvascular endothelial cells

**DOI:** 10.1186/2040-7378-3-12

**Published:** 2011-10-26

**Authors:** Pauline Patak, Fengyan Jin, Simon T Schäfer, Eric Metzen, Dirk M Hermann

**Affiliations:** 1 Department of Neurology, University Hospital Essen, Germany; 2 Department of Physiology, University of Duisburg-Essen, Germany; 3 Department of Anaesthesiology and Intensive Care Medicine, University Hospital Essen, Germany

**Keywords:** Blood-brain barrier, hypoxia-inducible factor, multidrug resistance, stroke

## Abstract

**Background:**

ATP-binding cassette transporters at the blood-brain barrier are actively regulated upon ischemic stroke in a way that impedes the access of pharmacological compounds to the brain tissue. The luminal endothelial transporter ABCB1 was recently shown to be increased, whereas the abluminal transporter ABCC1 was decreased on ischemic brain capillaries. *In vitro *studies using epithelial cells suggested that ABCB1 is regulated during hypoxia in a hypoxia-inducible factor (HIF)-1α-dependent way.

**Methods:**

In order to investigate whether hypoxia might be responsible for the expression changes of ABCB1 and ABCC1 in the ischemic brain, the immortalised human brain microvascular endothelial cell line hCMEC/D3 was exposed to hypoxia (1%) or anoxia (0%). Cell lysates were analysed by Western blot to detect the protein expression of ABCB1, ABCC1, HIF-1α and HIF-2α.

**Results:**

During hypoxia, an accumulation of HIF-1α and HIF-2α was noticed in hCMEC/D3 cells that followed different time kinetics. Both HIF-1α and HIF-2α abundance increased within 4 h of hypoxia. HIF-1α levels decreased to below detection levels within 16 h of hypoxia, whereas HIF-2α remained elevated even after 48 h. No changes of ABCB1 and ABCC1 expression were detected, neither on the mRNA nor protein level.

**Conclusion:**

Our data suggests that other factors than hypoxia may be responsible for the expression changes of ATP-binding cassette transporters in the ischemic brain.

## Background

ATP-binding cassette (ABC) transporters are efflux proteins that are abundantly expressed at the blood-brain barrier [1,2]. ABC transporters protect the brain from toxic compounds, but at the same time they prevent the access of drugs into the brain [1,2]. In brain disease, changes of ABC transporter expression and hence function have been demonstrated to modulate barrier properties [2]. Upon ischemia, ABC transporters are regulated on brain capillary endothelial cells in a coordinated way that impedes the delivery of drugs into the brain. As such, the luminal transporter ABCB1, which transfers its substrates from the brain into the blood, was increased [3], whereas the abluminal transporter ABCC1, which carries its substrates in the opposite direction, i.e. from blood to brain was decreased [4] following middle cerebral artery occlusion (MCAO) in mice. The altered abundance had a major impact on the biodistribution of drugs in the brain and resulted in a poorer delivery of neuroprotective drugs to the post-ischaemic brain, despite a stroke-related impairment of blood-brain barrier integrity [3,4].

Understanding the regulatory mechanisms of the luminal to abluminal ABC transporter balance is an important challenge for brain pharmacology, as it may identify strategies that improve the access of drugs to the brain. Interestingly, the expression of ABCB1 has previously been shown to be regulated in hypoxic epithelial cells in a hypoxia-inducible factor (HIF)-1α-dependent way [5]. Since hypoxia is a major factor contributing to ischemic injury, we were intrigued by the question whether HIF-1α-dependent signaling is also responsible for the ischemia-induced expression changes of ABC transporters in endothelial cells. To elucidate this issue, we exposed the cells of the immortalised human brain microvascular endothelial cell line hCMEC/D3 to conditions of ambient hypoxia.

## Methods

### Cell culture

hCMEC/D3 cells were propagated in Microvascular Endothelial Cell Medium-2 (EGM-2MV; obtained from Lonza, Allendale, NJ, U.S.A.) [6]. Cells from passage 25 to 35 were grown on surfaces coated with 100 μg/ml rat tail collagen type-I (BD Biosciences, Heidelberg, Germany). Cells were plated on collagen-coated 60 mm dishes and placed in a hypoxia chamber (oxygen concentration: 1%). In additional studies, HIF-1α was chemically induced by supplementing the cells with the iron chelator deferoxamine mesylate (DFO) (0.1 mM; Sigma-Aldrich, Deisenhofen, Germany) or the prolyl-4 hydroxylase inhibitor dimethyloxaloyl glycine (DMOG) (1 mM; Alexis Biochemicals, Lörrach, Germany). To generate oxygen concentrations close to anoxia, cells were furthermore incubated in GasPak (BBL) anoxic jars using BD GasPak EZ anoxic container systems (BD Diagnostic Systems, Heidelberg, Germany). The duration of hypoxia or anoxia exposure is specified below. All experiments were performed at least three times.

### Antibodies

The monoclonal antibody against human HIF-1α (610959) was purchased from BD Biosciences (Heidelberg, Germany). The polyclonal antibody against HIF-2α (AF 2886) was from R&D Systems (Wiesbaden-Nordenstadt, Germany). The polyclonal antibody against ABCB1 (sc-8313) was from Santa Cruz Biotechnology (Santa Cruz, CA, U.S.A.) and the monoclonal antibody against ABCC1 was purchased from Alexis Biochemicals (Lörrach, Germany). The polyclonal antibody against actin (A2103) was from Sigma-Aldrich. The goat polyclonal, horse radish peroxidase (HRP)-conjugated antibodies raised against rabbit (PO448) and mouse (PO447) IgG were purchased from DAKO (Hamburg, Germany).

### Western blots

Western blotting for ABCB1 and ABCC1 was performed as described previously [7]. Briefly, cell lysates were prepared using RIPA lysis buffer (50 mM Tris pH 7.5, 0.1% SDS, 1% Nonidet P40, 0.5% sodium deoxycholate, 2 mM EDTA, 150 mM NaCl) containing protease inhibitor cocktail (Roche, Mannheim, Germany). Protein samples were separated on 7.5% reducing SDS gels and blotted onto PVDF membranes. After the transfer, blocking of unspecific binding sites was achieved by incubation in Tris-buffered saline (50 mM Tris/HCl, 150 mM NaCl) containing 0.5% Tween 20 (TBST, pH 7.2) and 5% skimmed milk. Washed membranes were incubated overnight at 4°C with antibody against ABCB1 (1:1000 in TBST) or ABCC1 (1:200 in TBST) and for 2 h at 20°C with antibody against HIF-1α (1:1000 in TBST) and HIF-2α (1:1000 in TBST). Following incubation with HRP-conjugated secondary antibodies, the target proteins were detected with the enhanced chemoluminescence (ECL) kit (GE Healthcare, Munich, Germany) using an FX7 chemoluminescence documentation system (Peqlab, Erlangen, Germany).

### Real time quantitative PCR (rt-PCR)

Total RNA was extracted from hCMEC/D3 cells with RNeasy Midi Kit (Qiagen, Hilden, Germany) according to manufacturer's protocol. 1 μg RNA was reverse-transcribed to cDNA. The following *abcb1 *and *abcc1 *primers were used: *abcb1 *(84 bp): 5', AAA TTG GCT TGA CAA GTT GTA TAT GG; 3', CAC CAG CAT CAT GAG AGG AAG TC; *abcc1 *(139 bp): 5', TCT ACC TCC TGT GGC TGA ATC TG; 3', CCG ATT GTC TTT GCT CTT CAT G. In our studies, *succinate dehydrogenase *(*sdha*) was analysed as housekeeping gene using primers provided by Qiagen (cat.-no.: QT00059486). *Abcb1, abcc1 *and *sdha *mRNA expression was analysed using SYBR^® ^green as fluorescent dye (Eurogentec, Verviers, Belgium) on the Step One Plus Real Time PCR Detection System (Applied Biosystems, Darmstadt, Germany) in a two-step rt-PCR. The denaturation steps were performed at 95°C for 10 min followed by 40 cycles at 95°C for 15 s, and at 60°C for 1 min. Rt-PCR was always performed in duplicates, for which mean values were determined. Amounts of cDNA were normalized to *sdha *using the Δct method, as previously described [7].

## Results

### HIF-1α and HIF-2α expression in hCMEC/D3 cell line during normoxia and hypoxia

**Figure 1:**
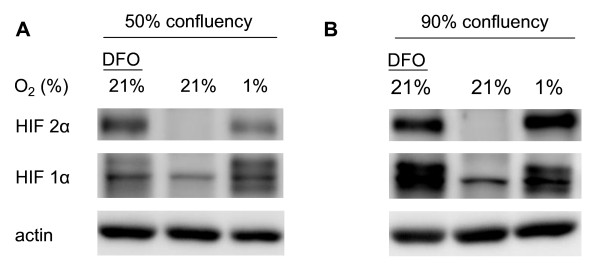
**Hypoxia-inducible accumulation of HIF-1α and HIF-2α in hCMEC/D3 cells depends on confluency state**. Cells were exposed to normoxia (21% oxygen) or hypoxia (1% oxygen) for 6 hours. For chemical HIF-1α and HIF-2α induction, normoxic cells were treated with 0.1 mM of the iron chelator deferoxamine mesylate (DFO). HIF-1α and HIF-2α abundance was examined using Western blot analysis.

To eludicate the impact of cell confluency on hypoxia-inducible expression of HIF-1α and HIF-2α, cells seeded at different densities were exposed to 1% oxygen for 6 hours and compared with control cells that were simultaneously kept under normoxia with or without the addition of 0.1 mM DFO. Western blot analysis revealed that whereas no HIF-1α and HIF-2α protein was detectable in control cells under normoxia, both hypoxia and DFO induced HIF-1α and HIF-2α accumulation in hCMEC/D3 cells (Figure 1A, B). Interestingly, HIF-1α and HIF-2α abundance changed with confluency state. As such, both the basal and hypoxic expression of both proteins significantly increased with increasing cell density (Figure 1A, B).

### Kinetics of HIF-1α and HIF-2α expression in hCMEC/D3 during hypoxia

**Figure 2:**
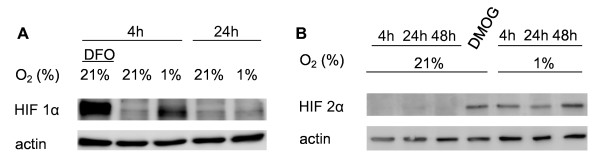
**HIF-1α is transiently induced whereas HIF-2α is continuously elevated in hypoxic hCMEC/D3 cells**. Cells were exposed to normoxia (21% oxygen) or hypoxia (1%) for various hours. For chemical HIF-1α and HIF-2α induction, 0.1 mM of DFO or 1 mM of the prolyl-4 hydroxylase inhibitor dimethyloxaloyl glycine (DMOG) for 4 h were administered. HIF-1α and HIF-2α abundance was assessed by Western blot analysis.

To evaluate the kinetics of hypoxic induction of HIF-1α and HIF-2α, hCMEC/D3 cells were exposed to different durations of hypoxia. Western blot analysis revealed that both proteins were increased when cells were exposed to 1% oxygen for 4 h (Figure 2A, B). Interestingly, HIF-2α levels continuously remained elevated after 24 and 48 h hypoxia (Figure 2B). In contrast, HIF-1α had declined again after 24 h of oxygen deprivation (Figure 2A). Cells exposed to 1 mM DMOG or 0.1 mM DFO for 4 h served as positive controls.

### ABCB1 and ABCC1 expression remains unchanged in hCMEC/D3 cells during hypoxia and anoxia

**Figure 3:**
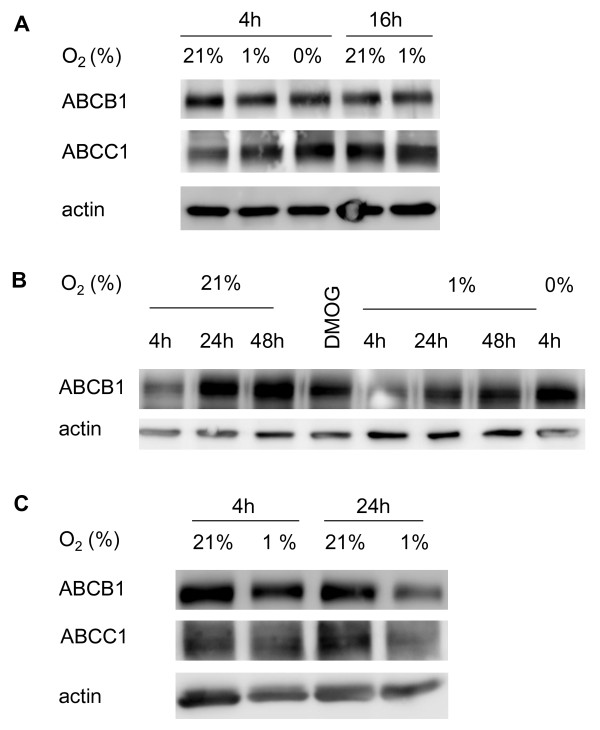
**ABCB1 and ABCC1 abundance is not influenced by hypoxia or anoxia in hCMEC/D3 cells**. Cells were exposed to normoxia (21% oxygen), hypoxia (1%) or anoxia (0%, in A) for 4 to 48 hours. The abundance of ABCB1 and ABCC1 protein was evaluated by Western blot analysis. As positive control, cells exposed to the prolyl-4 hydroxylase inhibitor dimethyloxaloyl glycine (DMOG) were assessed (in B). Note the absence of expression changes at any of the time points examined (in A-C).

**Figure 4:**
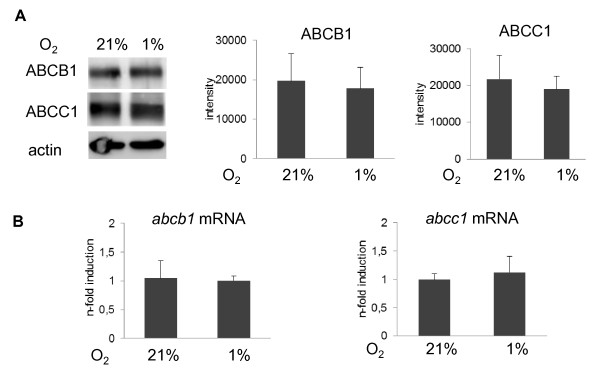
**ABCB1 and ABCC1 expression is not influenced by hypoxia on the protein and mRNA level**. Cells were exposed to normoxia (21% oxygen) or hypoxia (1%). The abundance of ABCB1 and ABCC1 protein was examined by Western blot analysis after 16 h (in A), whereas *abcb1 *and *abcc1 *mRNA levels were analysed by rt-PCR after 4 h exposure (in B). Data are means ± SD values. Results from four (in A) or three (in B) different experiments are shown. Rt-PCR data were normalised to *sdha *that was evaluated as housekeeping gene.

Cells exposed to 4 to 48 h hypoxia or 4 h anoxia were evaluated by Western blot analysis [7] for changes in the abundance of the ABCB1 and ABCC1 protein. In addition, cells exposed to 4 h hypoxia were assessed by rt-PCR for changes in *abcb1 *and *abcc1 *mRNA expression. In none of the experimental conditions examined, changes in ABCB1 or ABCC1 protein or *abcb1 *and *abcc1 *mRNA levels were found (Figures 3 and 4).

## Discussion

We report that ABC transporters are not regulated by hypoxia in the immortalised human brain microvascular endothelial cell line hCMEC/D3, which provides a unique and accessible *in vitro *system to study protein responses during sustained oxygen deprivation [6]. Our interest was sparked by *in vivo *studies in a model of MCAO that demonstrated an increase of ABCB1 [3] and a decrease of ABCC1 [4] protein levels on the surface of ischemic cerebral microvessels. Studies in the epithelial cell lines OKF6, T84 and Caco-2 had shown that the ABCB1 transporter was regulated in a HIF-1α-dependent way upon hypoxia [5]. Thus, we hypothesized that ABCB1 expression in human brain microvascular endothelial cells may also be enhanced as a consequence of HIF activation.

In our study, both HIF-1α and HIF-2α were found to be induced upon hypoxia. While HIF-2α exhibited a long-lasting accumulation in the brain endothelial cell line, HIF-1α levels were only transiently increased. While HIF-1α is known to be expressed in virtually all tissues, HIF-2α has been shown to be restricted to certain cell types including endothelial cells, cells of the kidney, liver, lungs and pancreas [8]. However, no changes in ABCB1 or ABCC1 protein levels were detected in our present study, despite a strong induction of HIF-1α and HIF-2α in the hCMEC/D3 cells, suggesting that HIF might not be essential for ABC transporter regulation in cerebral microvessels. The regulation of both transporters seen in cerebral ischaemia could depend on other factors than hypoxia alone that go hand in hand with ischaemia and have been described to induce ABCB1 expression, such as glucose depletion [9] or reactive oxygen species that are generated by reoxygenation as shown in rat brain endothelial cells [10,11].

To our knowledge only three other studies have so far examined the effect of hypoxia on ABC transporters in brain endothelial cells. In a primary rat brain endothelial cell model of hypoxia/reoxygenation, either induced by hydrogen peroxide administration or by reoxygenation following 6 hours of sustained hypoxia, Felix and Barrand [10] showed increased levels of ABCB1 6 hours following hypoxia and then again 24 hours following reoxygenation. Robertson et al. [11] studied the expression of ABCB1 in primary and immortalised rat brain endothelial cells that underwent hypoxia/reoxygenation. Exposure of primary rat brain endothelial cells to 6 hours of hypoxia alone revealed no changes in ABCB1 protein levels compared to normoxic controls. Significant increases in ABCB1 levels were only observed 24 h after 6 h hypoxia with reoxygenation. Xiao-Dong et al. [12] observed that repetitive hypoxia/reoxygenation may induce an up-regulation of ABCB1 in cultured rat brain microvascular endothelial cells. In that study, cells underwent hypoxia by covering the cells with paraffin oil once daily for 15 min over a time period of 8 days. None of the authors so far investigated the effect of over-night exposure to hypoxia on ABCB1 levels.

We did not investigate reoxygenation conditions in the present study, since we were interested in the role of HIF-1α and HIF-2α in the regulation of ABC transporters. Neither did we investigate ABC transporter expression in co-culture with human astrocytes that are known to have a regulatory function at the blood-brain barrier [13]. Based on the data presented here, it seems that the responses of ABC transporters to hypoxia are less distinct than previously presumed. This is underlined by recent observations in prostate carcinoma cells, showing that the protein expression of ABCB1 and ABCC1 were independent of tissue oxygen levels [14]. Our results support the idea that the mechanisms controlling the expression of ABCB1 and ABCC1 are cell specific. Thus, hypoxia does not seem to play a major role in the regulation of ABC transporters in cerebral microvessels.

## Conclusion

This is the first study examining the expression of HIF and ABC transporters under sustained hypoxia in a human cell model of the blood-brain barrier. Despite a strong induction of HIF-1α and HIF-2α, this study did not detect a regulation of the transporters ABCB1 and ABCC1, indicating that neurovascular signals other than HIF-1α and HIF-2α are responsible for the expression changes of ABC transporters after MCAO.

## Competing interests

The authors declare that they have no competing interests.

## Authors' contributions

PP carried out the cull culture experiments and Western blots and drafted the manuscript. FJ assisted with the Western blots and helped to analyse the data. SS performed the rt-PCR experiments. EM and DMH designed the study, evaluated the data and corrected the draft. All authors read and approved the manuscript.
